# Efficacy and safety of two tip flexible suctioning ureteral access sheaths combined with a 7.5Fr flexible ureteroscope: a retrospective study

**DOI:** 10.3389/fsurg.2025.1628264

**Published:** 2025-08-07

**Authors:** Jinquan Luo, Yueming Li, Jielin Lin, Xinyi Li, Wang He, Runqiang Yuan, Mancheng Gong

**Affiliations:** ^1^ Department of Urology, The People’s Hospital of Zhongshan, Zhongshan, Guangdong, China; ^2^ Department of Urology, Sun Yat-sen Memorial Hospital, Guangzhou, Guangdong, China

**Keywords:** retrograde intrarenal surgery, ureteral access sheath, flexible ureteroscopy, stone-free rate, suctioning

## Abstract

**Objective:**

This study aimed to compare the efficacy and safety of two tip-flexible suctioning ureteral access sheaths (TFS-UAS), 12/14Fr (Group A) and 10/12Fr (Group B), combined with a 7.5Fr flexible ureteroscope (f-URS) for treating upper ureteral and renal stones.

**Methods:**

A retrospective analysis of 168 patients undergoing retrograde intrarenal surgery (RIRS) was conducted. Group A (*n* = 76) used a 12/14Fr TFS-UAS, while Group B (*n* = 92) used a 10/12Fr TFS-UAS. Primary outcomes included stone-free rates (SFR) (Grade I: ≤2 mm; Grade II: ≤4 mm fragments), operative times, and complications.

**Results:**

Baseline characteristics were comparable between groups. Group A demonstrated significantly shorter operation (72.5 vs. 78.5 min, *P* < 0.05) and lithotripsy durations (57.5 vs. 64.5 min, *P* < 0.05). Immediate SFR (Grade I) was higher in Group A (86.8% vs. 71.7%, *P* = 0.018), but 1-month SFR showed no difference (90.8% vs. 84.4%, *P* = 0.242). Grade II SFR and complication rates (ureteric injury, fever, sepsis) were similar (*P* > 0.05).

**Conclusion:**

The 12/14Fr TFS-UAS combined with a 7.5Fr f-URS offers superior lithotripsy efficiency and immediate SFR compared to the 10/12Fr variant, while maintaining comparable long-term outcomes and safety. These findings support its clinical preference for RIRS in upper tract calculi.

## Introduction

Urolithiasis significantly endangers patient health, with prevalence rates ranging from 1% to 20% globally (1). The European Association of Urology guidelines indicate that for intermediate renal stones and proximal ureteral stones exceeding 1 cm, the flexible ureteroscope (f-URS) is one of the most effective treatment options available (2).

Recently, the introduction of the tip-flexible suction ureteral access sheath (TFS-UAS) has led to notable changes in the treatment approach for upper urinary tract stones, achieving high stone-free rates (SFR) along with minimal severe adverse events and reintervention rates (3). Importantly, both the size of the TFS-UAS and the f-URS play a crucial role in determining SFR and safety outcomes. Current research suggests that the 10/12Fr TFS-UAS offers better SFR compared to the 12/14Fr counterpart while maintaining similar safety metrics (4, 5). However, Hu et al. found that employing a 7.5 Fr f-URS in conjunction with Fr12/14 TFS-UAS for kidney stone treatment yields greater lithotripsy efficiency and a reduced complication rate, as opposed to using the 9.2 Fr f-URS with traditional f-UAS (6). As such, exploring the combination of these two types of TFS-UAS with smaller-caliber flexible ureteroscopes is necessary to enhance clinical outcomes.

This study intends to assess and contrast the effectiveness and safety of two variations of TFS-UAS when paired with a 7.5Fr flexible ureteroscope for the management of upper ureteral and renal stones.

## Patients and methods

A retrospective analysis was performed involving 168 patients suffering from upper ureteral or renal stones who underwent retrograde intrarenal surgery (RIRS) at the Department of Urology in The People's Hospital of Zhongshan between October 2,024 and February 2025. The study aimed to evaluate the outcomes of RIRS based on different sizes of the TFS-UAS employed during the procedure. To facilitate this analysis, the patients were categorized into two distinct groups according to the diameters of the TFS-UAS utilized: Group A and Group B. In Group A, the surgical intervention was carried out using a 7.5 Fr flexible ureteroscope (model: PU3033A) paired with an F12/14 TFS-UAS. Conversely, in Group B, the same model of the flexible ureteroscope was used, but alongside an F10/12 TFS-UAS for the RIRS procedure. Prior to the commencement of the study, all patients were informed about the nature and purpose of the research, and consented to participate, ensuring an ethical approach to the investigation. All patients signed informed consent forms prior to participating in the study, and this study received approval from the Ethics Committee of The People's Hospital of Zhongshan, reinforcing the commitment to adhere to ethical standards in clinical research.

All patients received preoperative non-contrast computed tomography (CT) scans, which were conducted to thoroughly examine the characteristics of their calculi. The size of the calculi was determined by measuring the maximum diameter, with particular attention paid to the largest stone in patients who had multiple stones present. Furthermore, the stone density was assessed based on the Hounsfield Unit (HU) values provided in the radiologist's CT report. The total operation time for each patient was rigorously recorded, beginning from the initial insertion of the ureteroscope and concluding with the successful completion of urethral catheterization. The duration of lithotripsy was defined as the entire period during which the holmium laser was in contact with the calculi until the f-URS was removed.

The primary outcomes of this study included the assessment of immediate and one-month postoperative SFR, as well as the complication rates experienced by the patients. A patient was classified as having achieved stone-free status if follow-up evaluations using ultrasonography or standard radiography indicated no residual calculi or only small fragments. Specifically, the criteria for grading SFR were established as follows: Grade I indicated the absence of residual calculi or only fragments that were 2 mm or smaller, while Grade II allowed for fragments up to 4 mm in size. This grading system aligns with the guidelines set forth by the European Association of Urology, which designates residual fragments of 4 mm or smaller as insignificant due to their lower likelihood of leading to notable clinical complications and their higher probability of being expelled spontaneously (7). Moreover, fragments measuring 2 mm or smaller are particularly noteworthy because they exhibit decreased chances of growth, along with increased rates of spontaneous passage and reduced incidences of complications and the need for subsequent interventions (8). In instances where SFR was not achieved, there are potential treatment pathways, which may include options such as extracorporeal shock wave lithotripsy (ESWL) or a second procedure involving RIRS.

For the evaluation of intraoperative ureteral mucosal injuries, the criterion hinges on direct observation during f-URS, specifically noting the occurrence of mucosal rupture that exposes underlying smooth muscle (9). Additionally, the diagnosis of postoperative urosepsis was made in accordance with the Third International Consensus Definitions for Sepsis and Septic Shock (10), ensuring that a standardized approach was utilized in identifying and managing this complication.

The criteria for inclusion in this study were defined as follows: a confirmed diagnosis of upper ureteral or renal stones, identified through CT imaging, with a longest diameter between 1.0 and 3.0 cm; absence or control of urinary tract infections; participants must be older than 18 years; no history of spinal deformities or ureteral stenosis; and providing voluntary consent for RIRS. Exclusion criteria encompassed: acute urinary tract infections; pregnancy or breastfeeding; compromised cardiopulmonary health; states of hypercoagulability or irregular cardiac or pulmonary conditions; the presence of concurrent tumors; prolonged use of oral glucocorticoids; and any other surgical contraindications.

**Figure 1:**
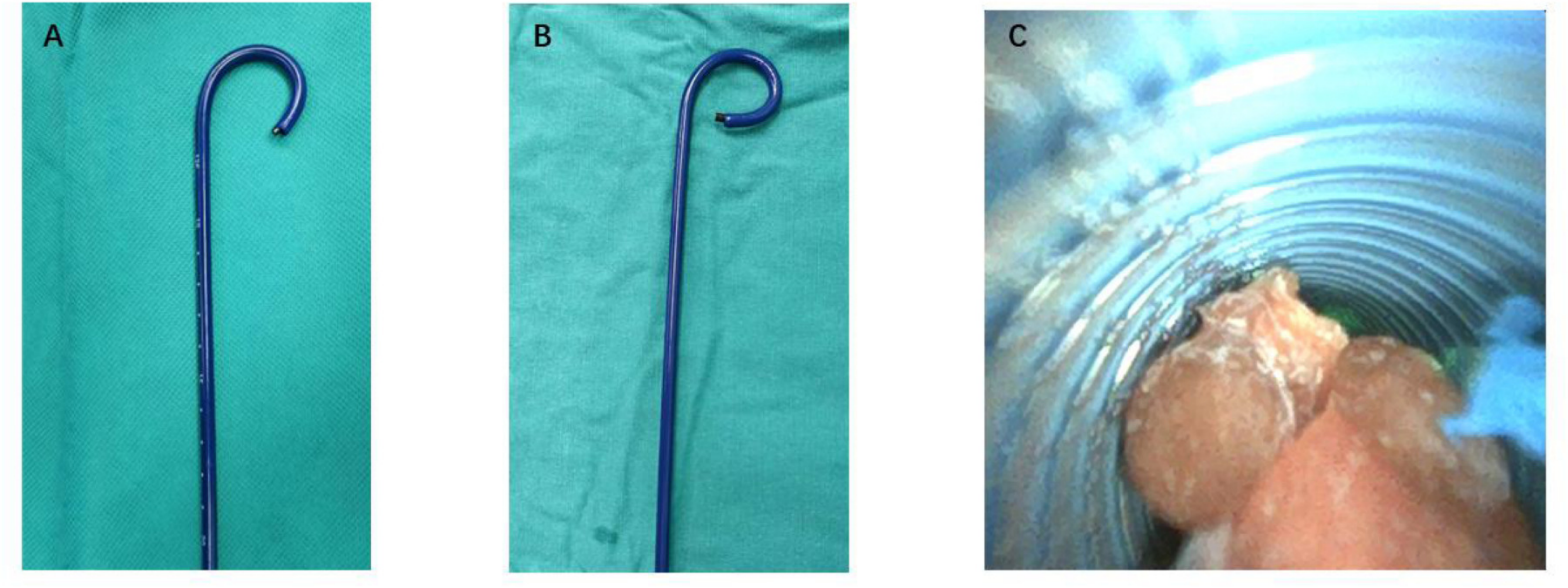
*in vitro* demonstration of the 7.5Fr ultra-thin flexible ureteroscope combined with two types of TFS-UAS and negative-pressure stone extraction. **(A)** The 7.5Fr flexible ureteroscope paired with a 12/14Fr TFS-UAS at maximum deflection. **(B)** The 7.5Fr flexible ureteroscope paired with a 10/12Fr TFS-UAS at maximum deflection. **(C)** Negative-pressure stone extraction using the 12/14Fr TFS-UAS. TFS-UAS, tip-flexible suctioning ureteral access sheaths.

Under general anesthesia, the patient was placed in the lithotomy position, following standard disinfection and draping, using either endotracheal intubation or a laryngeal mask airway. A ureteroscope was utilized to examine the ureter, and any double-J stents placed prior to surgery were removed. Upon access to the upper ureter, a zebra guidewire was retained. A TFS-UAS (UAS-Q−1236/1246, Opper Medical, Guangdong, China) of either 12/14 Fr (Figure 1A) or 10/12 Fr (Figure 1B) (46 cm for males, 36 cm for females) was inserted under the guidance of the zebra without fluoroscopic guidance. This was then linked to a negative pressure suction apparatus, which functioned within a negative pressure range of −20 to −5 kPa. After insertion of a single-use 7.5 Fr flexible ureteroscope (PU3033A, Pusen Medical, Guangdong, China), lithotripsy was performed using a 272 μm holmium laser fiber, calibrated to 20–40 W (1.0–2.0 J × 20 Hz). Normal saline was employed as the irrigation fluid, administered through an irrigation pump. Within Group A, the irrigation pressure was sustained between 100 and 150 mmHg at a flow rate of 300–400 ml/min, while in Group B, the pressure was adjusted to 100–120 mmHg with a flow rate of 100–200 ml/min. In both groups, a constant negative pressure was upheld throughout the lithotripsy procedure. Larger fragments were directly aspirated (Figure 1C), whereas smaller debris was removed via the gap between the flexible ureteroscope and the ureteral access sheath. A ureteral stent was placed in all patients after the surgery.

Statistical analysis was conducted utilizing SPSS version 27.0. Qualitative variables such as gender, prior placement of a double-J stent before surgery, the rate of surgical success, and postoperative SFR were expressed in terms of rates or proportions. To assess differences in these variables across various groups, Fisher's exact test and the chi-square test (*χ*^2^) were applied, with a *P* value of less than 0.05 considered indicative of a statistically significant difference. Age, dimensions of calculi, duration of the operation, and lithotripsy time displayed a skewed distribution and were summarized using the median (M), along with the first (Q1) and third quartiles (Q3). The Mann–Whitney *U* test was employed to evaluate differences in these metrics between different groups, where a *P* value under 0.05 signified a statistically significant difference.

## Results

In the study, 168 patients were analyzed, consisting of 76 individuals in Group A (44 males and 32 females) with a median age of 51 years (interquartile range: 42–60). Group B included 92 patients (52 males and 40 females), with a median age of 53 years (interquartile range: 44–61). Comprehensive characteristics can be found in Table 1. The baseline features of both groups were similar, indicating no significant variations in median age, gender ratio, prevalence of hypertension and diabetes, clinical presentations, urine leukocyte counts, urine nitrite levels, and preoperative urine cultures. The characteristics of the stones, including their locations and diameters, as well as the median Hounsfield units, were also found to be alike in both groups, with Group A exhibited a median stone diameter of 13 mm and a median Hounsfield unit of 1299, whereas Group B demonstrated a median stone diameter of 14 mm and a median Hounsfield unit of 1,192 (*p* = 0.160, *p* = 0.100, respectively).The rate of pre-stenting in patients was similarly distributed across both groups (52.6% vs. 40.2%, *p* = 0.842).

**Table 1:** Demographic characteristics and baseline data of two groups.

Parameter/Characteristic	Group A (*n* = 76)	Group B (*n* = 92)	*P*-value
Age	51 (42,60)	53 (44,61)	0.404
Male gender	44 (57.9)	52 (56.2)	0.858
BMI, kg/m²	25 (22,27)	24 (22,27)	0.273
Hypertension	24 (31.6)	41 (44.6)	0.085
Diabetes	12 (15.8)	14 (15.2)	0.919
Presentation			
Pain	58 (76.3)	72 (78.2)	0.764
Gross hematuria	8 (10.5)	11 (12.0)	0.771
Fever	5 (6.6)	2 (2.2)	0.301
Incidental	5 (6.6)	7 (7.6)	0.796
Urine leukocyte (/HP)			
Normal	18 (23.7)	28 (30.4)	0.329
+	16 (21.1)	26 (28.2)	
++	22 (28.9)	19 (20.7)	
+++	20 (26.3)	19 (20.7)	
Urine nitrite (Positive)	9 (11.8)	9 (9.8)	0.352
Preoperative urine culture positive	17 (22.4)	14 (15.2)	0.234
Blood white cell count (>9.5*10^9 ^/L)	14 (18.4)	10 (10.9)	0.164
Serum creatinine (umol/L)	77 (66,88)	71 (59,86)	0.192
Pre-stenting	40 (52.6)	47 (40.2)	0.842
Maximum diameter of calculus (mm)	13 (10,16)	14 (11,17)	0.160
Diameter of calculus between 20 mm–30 mm	11 (14.5)	12 (13.0)	0.788
Stone Hounsfield unit (HU)	1,299 (1,072,1,472)	1,192 (969,1,396)	0.100
Stone location			0.134
Upper/middle pole	24 (31.6)	37 (40.2)	
Lower pole	22 (28.9)	29 (31.5)	
Proximal ureter and renal pelvic	30 (39.5)	26 (28.3)	

Regarding the operative characteristics and postoperative outcomes (Table 2), the operation duration for Group A was 72.5 min (range: 50–95), and the lithotripsy duration was 57.5 min (range: 35–77). In contrast, Group B had an operation duration of 78.5 min (range: 60–107) and a lithotripsy duration of 64.5 min (range: 46–91). The differences observed were statistically significant (*P* < 0.05). According to the standard Grade I, the immediate postoperative stone-free rate (SFR) was 86.8% (66/76) for Group A and 71.7% (66/92) for Group B, with a statistically significant difference (*P* = 0.018). The one-month postoperative SFR was 90.8% (69/76) for Group A and 84.4% (78/92) for Group B, indicating no significant difference between the two groups (*P* = 0.242). Based on the standard Grade II, the immediate postoperative SFR was 89.5% (68/76) for Group A and 81.5% (75/92) for Group B, with no statistically significant difference (*P* = 0.192). The one-month postoperative SFR was 93.4% (71/76) for Group A and 89.1% (82/92) for Group B, showing no significant difference between the two groups (*P* > 0.05). Postoperative complication rates were low, with a similar incidence of ureteric injury, fever, and sepsis observed between the groups.

**Table 2:** Operative characteristics and postoperative outcomes.

Outcome	Group A (*n* = 76)	Group B (*n* = 92)	*P*-value
Total operation time, min	72.5 (50,95)	78.5 (60,107)	**0** **.** **039**
lithotripsy time, min	57.5 (35,77)	64.5 (46,91)	**0**.**030**
Immediate postoperative SFR			
Grade I	66 (86.8)	66 (71.7)	**0**.**018**
Grade II	68 (89.5)	75 (81.5)	0.192
One-month postoperative SFR			
Grade I	69 (90.8)	78 (84.8)	0.241
Grade II	71 (93,4)	82 (89.1)	0.332
Ureteric injury			
Traxer low grade	5 (6.6)	3 (3.3)	0.521
Traxer high grade	0	0	N/A
Type of complications			
Fever	0	3	0.316
Urosepsis	0	0	N/A
Stone street formation	0	0	N/A
Hematuria needing intervention	0	0	N/A

The bolded values indicate postoperative outcomes with statistical significance.

SFR, stone-free rates.

## Discussion

Retrograde intrarenal surgery (RIRS) offers notable benefits in treating kidney stones, including its minimally invasive approach, impressive safety profile, and quick recovery after surgery. In recent years, the increased use of disposable electronic flexible ureteroscopes has effectively solved issues linked with traditional flexible ureteroscopes, such as high expenses, vulnerability to damage, maintenance difficulties, complex disinfection processes, and the risk of cross-infection (11, 12). Presently, the typical diameter for flexible ureteroscopes (f-URS) measures at 9.2 French (Fr) or slightly thinner. When paired with a 14 Fr ureteral access sheath, the narrow space between the scope and the inner wall of the sheath may limit the flow of irrigating fluid, leading to elevated intrarenal pelvic pressure (IPP) during the operation (13). This condition can result in postoperative issues like low back pain, infections, and even urosepsis (14), along with severe complications such as damage to the renal collecting system, rupture, and bleeding (15).

To manage IPP effectively during RIRS, the chief techniques currently utilized involve adjusting the pressure and flow rate of fluid irrigation, improving negative pressure suction, and reducing the diameter of the f-URS. Nevertheless, excessively low irrigation pressures and flow rates can result in issues such as reduced surgical visibility, prolonged operative durations, and considerable residual stone burdens. Therefore, decreasing the f-URS diameter along with enhanced negative pressure suction seems to present more practical benefits. Studies suggest that during RIRS, the relationship between the f-URS and the ureteral access sheath should follow the essential guideline of maintaining a ratio of endoscope to sheath diameter (RESD) of ≤0.75 to ensure safe IPP (16). Additionally, advanced pressure control systems (17, 18) and a range of different methods have been created to manage the IPP, aiming to reduce the risk of severe infections and other associated complications.

The ultra-fine 7.5 Fr flexible ureteroscope has progressively been adopted in clinical settings, and relevant investigations have verified its safety and reliability (19). This study's findings reveal that Group A exhibited superior performance compared to Group B concerning the duration of the operation and lithotripsy, in addition to achieving a higher immediate postoperative SFR. The primary factor contributing to the differences noted between the two groups is that the ureteral access sheath channel in Group A is wider than that in Group B, offering increased space for the vacuum-assisted extraction of fragmented stones. Moreover, the reduced RESD in Group A promoted the backflow of irrigation fluid, facilitating the concurrent expulsion of larger stone fragments through the space between the f-URS and the sheath. As a result, Group A not only enhanced the stone clearance rate but also shortened the operative time, significantly boosting the efficiency of lithotripsy.

The average duration of surgery was recorded at 72.5 min for Group A and 78.5 min for Group B, which exceeds the time frames noted in similar research (3, 4, 6). All ureteral access sheaths utilized in our investigation were positioned without fluoroscopic assistance, primarily due to historical constraints related to equipment and existing surgical methodologies. While the surgeons participating in the study possessed considerable expertise in RIRS and had undergone specialized fellowship training in endourology, we recognize that there is a learning curve for proficiently manipulating the sheath and scope. The FANS technique introduces a fresh approach, necessitating that the surgeon carefully guides the sheath to the targeted calyx while periodically retracting the scope to the Y junction for fragment removal. Additionally, our laser lithotripter, which has been operational for several years, may demonstrate a certain level of energy loss.

Proper irrigation during surgery is vital for maintaining a clear field and optimal working environment. Nonetheless, applying excessive irrigation pressure can lead to the backward flow of bacteria or endotoxins into the bloodstream through the renal tubules or veins, significantly contributing to infections after surgery. Furthermore, elevated irrigation pressure may precipitate low back pain and other postoperative symptoms (14, 16, 20). In Group A, when the perfusion pressure and the flow rate were adjusted to align with those of Group B, the reduced diameter of the RESD combined with continuous low negative pressure often resulted in a marked collapse of the renal pelvis. This issue complicated the lithotripsy process and increased the chances of mucosal injury and bleeding in the renal pelvis. After numerous adjustments and optimizations to ensure adequate distension of the renal pelvis, the ideal parameters were determined to be a perfusion pressure of 100–150 mmHg and a flow rate ranging from 300 to 400 ml/min. Under these circumstances, the renal pelvis typically remained only slightly collapsed, which helped maintain low intrarenal pressure (IPP) while enabling continuous suction of debris and small stone fragments. As a result, this method contributed to a clearer surgical environment and a decrease in postoperative complications, including fever, sepsis, and significant bleeding. Thus, while Group A necessitated higher perfusion pressures and flow rates in comparison to Group B, the synergy of continuous negative-pressure suction with a broader clearance channel for stones effectively sustained lower IPP, leading to fewer complications post-surgery, without any notable adverse effects.

One constraint of the current investigation is its design, which is not randomized. Additionally, the fact that this is a single-center study limits the generalizability of our findings. Consequently, it is imperative to conduct a larger, multicenter study that includes surgeons with a range of expertise levels to confirm the results of our investigation.

In summary, employing a 7.5 Fr f-URS in conjunction with 12/14Fr TFS-UAS for addressing upper ureteral and renal stones shows enhanced lithotripsy efficiency when compared to the the 10/12Fr variant, all while upholding similar safety standards. These findings could offer urologists evidence-based insights for choosing the most suitable UAS configuration for clinical use.

## Data Availability

The raw data supporting the conclusions of this article will be made available by the authors, without undue reservation.
